# Water diffusion in atmospherically relevant α-pinene secondary organic material†
†Electronic supplementary information (ESI) available. See DOI: 10.1039/c5sc00685f



**DOI:** 10.1039/c5sc00685f

**Published:** 2015-06-04

**Authors:** Hannah C. Price, Johan Mattsson, Yue Zhang, Allan K. Bertram, James F. Davies, James W. Grayson, Scot T. Martin, Daniel O'Sullivan, Jonathan P. Reid, Andrew M. J. Rickards, Benjamin J. Murray

**Affiliations:** a School of Earth and Environment , University of Leeds , Leeds , LS2 9JT , UK . Email: eehcp@leeds.ac.uk ; Email: b.j.murray@leeds.ac.uk ; Tel: +44-(0)113-343-9085 ; Tel: +44(0)-0113-343-2887; b School of Physics and Astronomy , University of Leeds , Leeds , LS2 9JT , UK; c School of Engineering and Applied Sciences , Harvard University , Cambridge , MA 02138 , USA; d Department of Chemistry , University of British Columbia , Vancouver , BC , Canada V6T 1Z1; e School of Chemistry , University of Bristol , Bristol , BS8 1TS , UK; f Department of Earth and Planetary Sciences , Harvard University , Cambridge , MA 02138 , USA

## Abstract

Secondary organic material (SOM) constitutes a large mass fraction of atmospheric aerosol particles. Understanding its impact on climate and air quality relies on accurate models of interactions with water vapour. Recent research shows that SOM can be highly viscous and can even behave mechanically like a solid, leading to suggestions that particles exist out of equilibrium with water vapour in the atmosphere. In order to quantify any kinetic limitation we need to know water diffusion coefficients for SOM, but this quantity has, until now, only been estimated and has not yet been measured. We have directly measured water diffusion coefficients in the water soluble fraction of α-pinene SOM between 240 and 280 K. Here we show that, although this material can behave mechanically like a solid, at 280 K water diffusion is not kinetically limited on timescales of 1 s for atmospheric-sized particles. However, diffusion slows as temperature decreases. We use our measured data to constrain a Vignes-type parameterisation, which we extend to lower temperatures to show that SOM can take hours to equilibrate with water vapour under very cold conditions. Our modelling for 100 nm particles predicts that under mid- to upper-tropospheric conditions radial inhomogeneities in water content produce a low viscosity surface region and more solid interior, with implications for heterogeneous chemistry and ice nucleation.

## Abstract 2

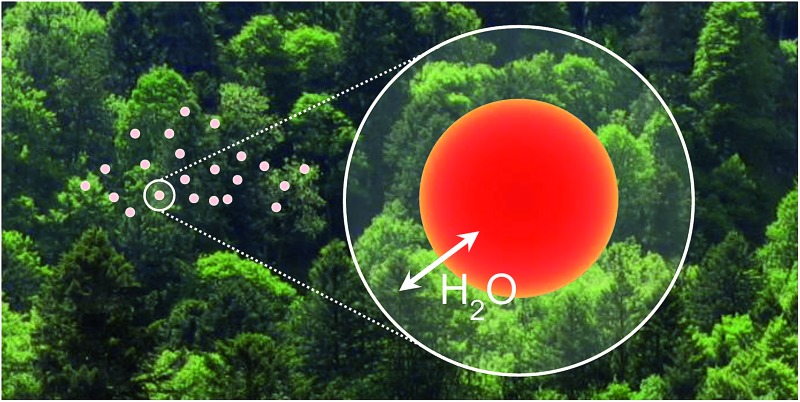
We report the first direct measurements of water diffusion coefficients in secondary organic aerosol.

## 

Atmospheric aerosol particles have an important impact on climate through their influence on cloud properties and their abilities to scatter and absorb radiation.^1,2^ Organic material constitutes a large fraction of the mass concentration of atmospheric aerosol particles,^3^ and a major source of condensed-phase organics in the atmosphere is the oxidation of volatile organic compounds (VOCs) to form secondary organic material (SOM) in the particle phase.^4^ Despite recent progress in the understanding of growth of SOM particles, discrepancies still exist between model descriptions and observations in mass yield and distribution.^5–8^

Recent research has shown that organic aerosol can be highly viscous or glassy at low relative humidity (RH) and/or low temperature.^9–13^ The presence of aerosol in this phase could have important implications for particle interactions with water vapour,^14^ condensed phase chemistry,^15^ lifetime and transport,^16^ as well as morphology^17^ and optical properties.^18^ It has been suggested that observed slow mixing and aerosol evaporation rates could be attributed to slow diffusion of the evaporating component within the particle bulk, imposing a kinetic limitation on the particle-to-gas evaporative flux.^19–22^ An alternative explanation, however, may be that the semi-volatile component can be of such low volatility (due to low vapour pressure and low mixing ratio) that the evaporative flux into the gas phase is low and the evaporation timescale is long, a process driven by the inherent thermodynamic properties of the complex mixture.^23^ Robinson, *et al.* ^24^ have observed mixing within laboratory-generated SOM on the minute timescale, and gas-particle partitioning occurred on a timescale of 1–2 hours in a study of pine-forest aerosol by Yatavelli, *et al.* ^25^ Rapid equilibration is also consistent with measurements that show SOM is an effective nucleus for cloud condensation with no evident kinetic delays.^26–29^ Detailed kinetic models^30–32^ are required to unravel the complexities of SOM processes, and these require knowledge of the diffusion coefficients of component species.

Highly viscous or glassy SOM may also nucleate ice in clouds.^33–35^ Proxies of SOM-containing aerosol, such as mixtures of carboxylic acids or organics, sugars and ammonium sulphate, are known to nucleate ice under cirrus conditions when in a highly viscous or glassy state.^36–39^ Ice nucleation by highly viscous or glassy aerosol has been suggested as an explanation for the presence of sulphate–organic material in cirrus ice crystal residues.^40^ Given that organic aerosol particles may play an important role in cirrus cloud formation, it is important to understand the phase of SOM across the full ranges of atmospherically relevant RH and temperature.

Water is often the most mobile component of highly viscous aqueous solutions^41^ and can act as a plasticiser.^14,42^ Measurements of water uptake and loss have previously been used to infer slow water diffusion coefficients in single-solute aqueous solutions such as sucrose.^42,43^ However, direct measurements of water diffusion in SOM do not currently exist. These are needed since proxy compounds like sucrose do not have the same properties as SOM.^44,45^ To address this, we report laboratory measurements of the water diffusion coefficient in water-soluble α-pinene SOM over a range of temperature and relative humidity conditions. The results are then parameterised and used in a multilayer spherical diffusion model to provide numerical simulations of water uptake and loss under atmospheric conditions. We find that water diffusion is not kinetically limited in 100 nm SOM particles on atmospheric timescales at 280 K, but becomes slow enough to impact atmospheric processes at lower temperatures.

## Results

### Diffusion measurements in α-pinene SOM

SOM was generated *via* the ozonolysis of α-pinene in a flow tube reactor, collected on a quartz filter and then the water-soluble component was extracted (or details of the method see ESI†). The experimental procedure for quantifying water diffusion coefficients in concentrated aqueous solutions has been described in detail previously.^46^ Briefly, for each experiment, a single aliquot of the SOM solution was pipetted onto a hydrophobic glass slide and formed into a disk of ∼200 μm radius and ∼25 μm thickness by placing a second, smaller glass slide on top, with spacers on either side. After equilibration in a temperature and humidity controlled cell to achieve a uniform water activity of RH/100, the H_2_O vapour in the gas flow was replaced by D_2_O vapour at the same dewpoint. D_2_O diffusion into the disk was observed *via* a Raman microscope, and the spatial and temporal evolution of the O–D and O–H bands were used to quantify water diffusion coefficients (see ESI† for details of the quantitative analysis of the SOM Raman spectra). Each Raman spectrum was acquired with a 514 nm laser using a 1 s exposure time and a laser power of 11 mW. The laser spot size was 1.3 μm, and a new position on the sample was probed for each spectral measurement, meaning that diffusion happened over a much larger scale than the size of the laser spot. The Raman spectrum of the SOM did not change – except for the O–H to O–D exchange – over the course of an experiment. This technique has been shown in the past to produce diffusion coefficients in good agreement with literature data for sucrose.^46^ Because the technique relies on the movement of an isotope, the measured diffusion coefficients are not influenced by the proton hopping mechanism (in which protons are transferred along a chain of water molecules in effect only, without the need for each individual proton to physically move from one end of the chain to the other).

Measurements were made between 240 and 280 K, over a water activity range of 0.15 to 0.8, and all results are listed in Table S2.† Experiments were repeated over a period of time to verify that the diffusion coefficients were unaffected by sample age. Measurements at lower temperatures were not possible due to the required duration of the experiments (data for the slowest diffusion coefficients presented here took several weeks to obtain) and measurements at higher temperatures were affected by an increase in sample fluorescence over the course of the experiment so are not reported.

**Fig. 1:**
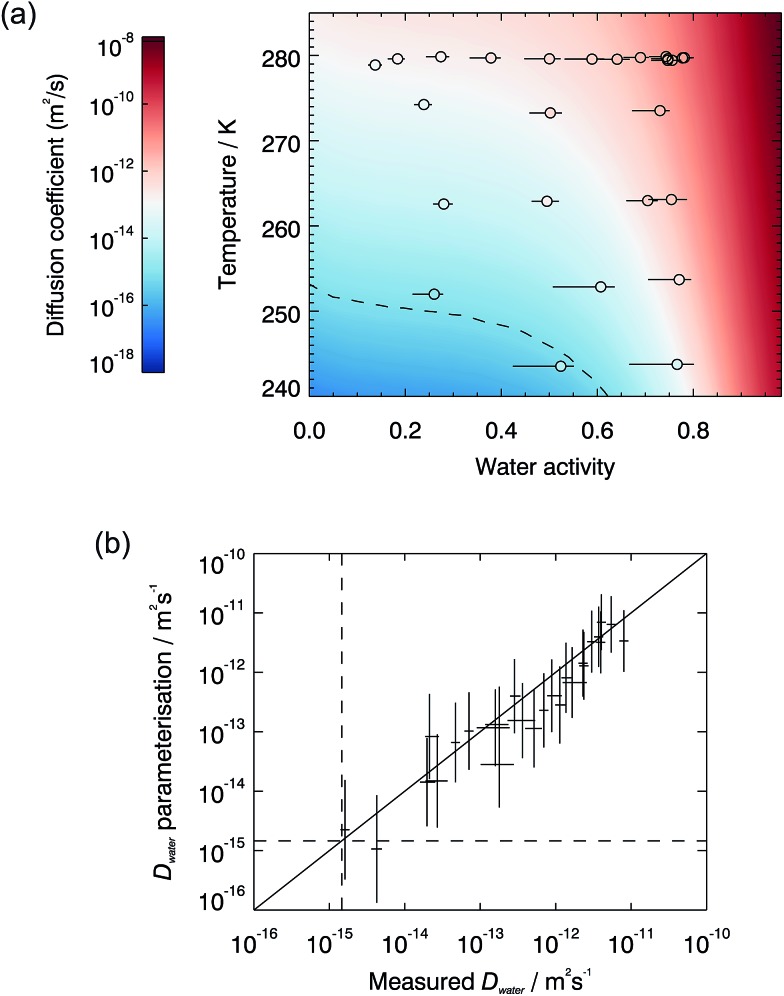
(a) Diffusion coefficients of water in SOM. Experimental data points are shown as coloured circles and the fit is the shaded background, both coloured on the same scale. The differences in colour between the background and circle interiors show the difference between the fit and the measured data points. Multiple measurements were performed at a water activity of 0.75 and 280 K over the course of 4 months to verify that the diffusive properties of the sample did not change with age. These datapoints are shown to be in good agreement with each other and the fit. (b) A one-to-one plot showing the measured water diffusion coefficients *vs.* the parameterisation. The dashed lines mark the regions above which the halftime for water diffusion into a spherical droplet of radius 100 nm is less than 1 s.

The measured diffusion coefficients are plotted *vs.* water activity and temperature in Fig. 1(a), with each data point representing a diffusion measurement on one disk. An empirical fit to the data was produced using a Vignes-type equation.^47^ This form of equation has been shown in the past to describe well the physical behaviour of the composition and temperature dependence of diffusion coefficients and be applicable in methanol/ethanol mixtures down to 100 K.^48^ It was used by Lienhard, *et al.* ^43^ at temperatures between 233 and 281 K to describe water diffusion in aqueous citric acid.

1 *D*_water_ = (*D*0water)^*x*_w_*α*^(*D*0SOM)^1–*x*_w_*α*^

where *D*0water is the temperature-dependent self-diffusion coefficient of water^49^ and *D*0SOM is the diffusion coefficient of water in amorphous SOM at a water activity, *a*_w_, of 0. *D*0SOM is constrained to fit the form of a Vogel–Fulcher–Tammann (VFT) relationship:^50^
2
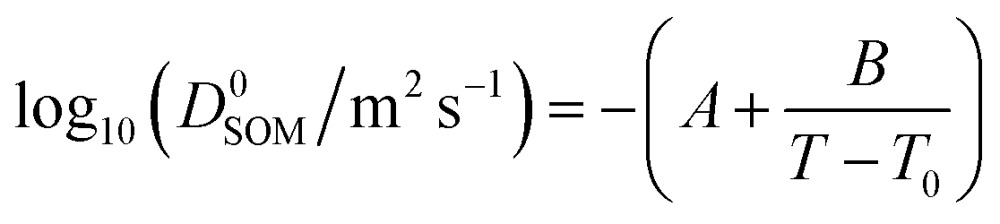
in which *A*, *B* and *T*_0_ are fitted parameters indicating the high temperature limit of the diffusion coefficient, the fragility and the temperature at which the diffusion coefficient would diverge, respectively. *x*_w_ is the mole fraction of water, calculated from the water activity using the effective hygroscopicity parameter (*k*_org_ = 0.1),^9,51^ and *α* is an activity coefficient.^43,52^ The fit is shown as the shaded surface in Fig. 1(a), and compared to the measured data points in a one-to-one plot in Fig. 1(b). Further details of the fitting procedure, together with the best fit parameters and their errors, are given in the ESI.†

**Fig. 2:**
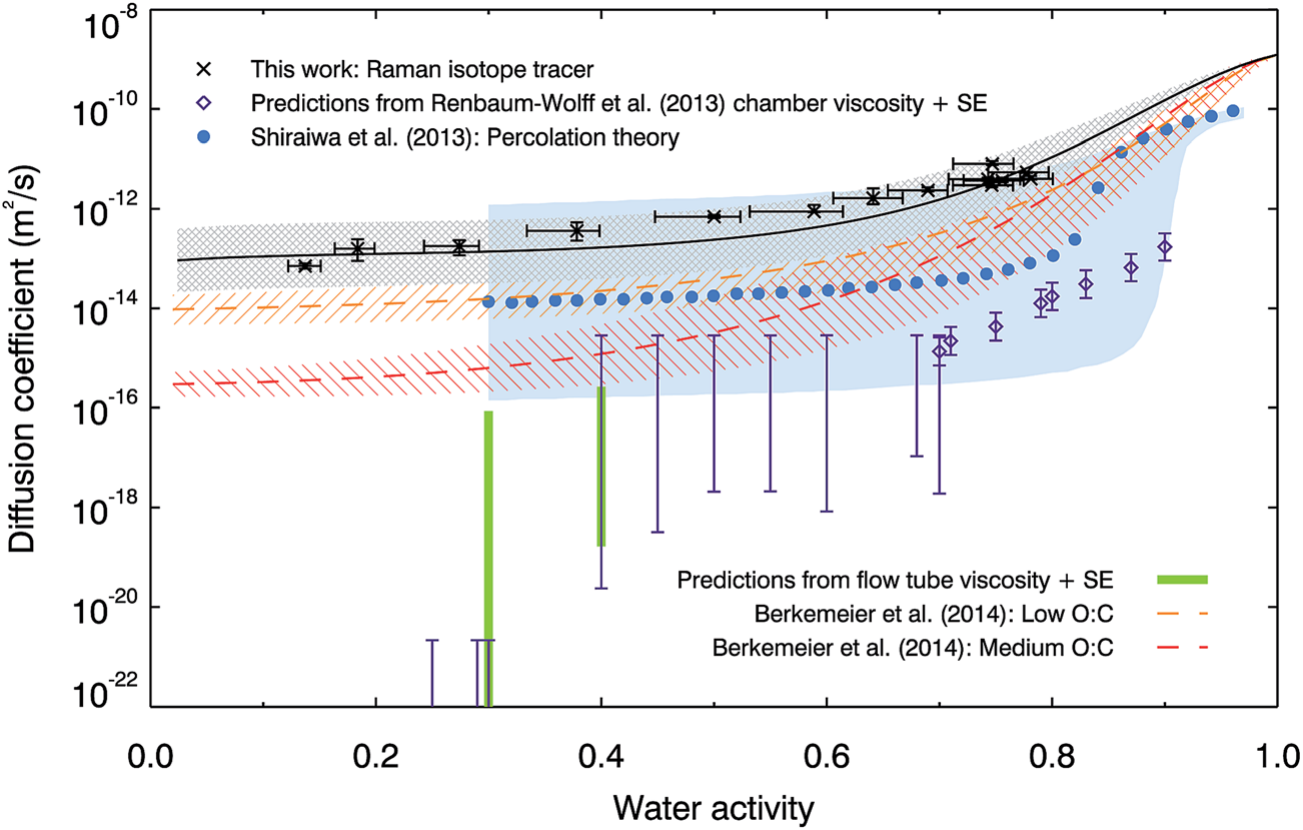
Comparison of measured diffusion coefficients of water in α-pinene SOM with literature data. Laboratory measurements at 280 K (black crosses, with our parameterisation shown as a black line, with grey shaded uncertainty region) are compared with predictions from percolation theory at room temperature (blue circles, with blue shaded uncertainty region^30^) and the semi-empirical method used by Berkemeier, *et al.* ^53^ for low (orange dashed line and hatched error region, O:C = 0.3) and medium (red dashed line and hatched error region, O:C = 0.5) oxidation states at 280 K. Also shown are the diffusion coefficients predicted by the Stokes–Einstein (S–E) equation with a hydrodynamic diameter of water of 2 Å, using the room temperature viscosity measurements on chamber-generated α-pinene SOM by Renbaum-Wolff, *et al.* ^11^ (generated using 80–100 ppb α-pinene and 300 ppb ozone, purple diamonds and bars), and on flow tube-generated α-pinene SOM (generated using 5 ppm α-pinene and 12 ppm ozone, green bars).

Fig. 2 compares our measured water diffusion coefficients at 280 K with predictions of water diffusion coefficients in α-pinene SOM based on the semi-empirical VFT-based approach by Berkemeier, *et al.*,^53^ also at 280 K. The predictions from this approach are lower than our experimental data; this could be due to differences between the composition of our SOM sample and the model SOM composition used by Berkemeier, *et al.* ^53^ in the semi-empirical VFT approach. We used only the water soluble component of SOM and it is possible that insoluble components could affect its viscosity or hygroscopicity, in turn affecting diffusive properties, however water-soluble material represents the major fraction of α-pinene SOM.^11^ We also compare our data with diffusion coefficients estimated from room temperature viscosity measurements made on α-pinene SOM generated in a chamber^11^ and in the flow tube (generated in a very similar manner to the SOM used in the diffusion experiments to facilitate a more direct comparison, for further details see ESI†). The Stokes–Einstein (S–E) equation is used here to convert viscosities to diffusion coefficients using a hydrodynamic diameter for water of 2 Å, but we find that it under-predicts the diffusion values for all conditions studied. The breaking down of the S–E relation at high viscosities is well known and the diffusion coefficients of small and large molecules have been shown to deviate near the glass transition in sugars^54–57^ and in protein.^58^ However, the magnitude of the observed deviations across the water activity range is remarkable. Below water activities of ∼0.3, the diffusion coefficients predicted by the use of the S–E equation are at least 8 orders of magnitude smaller than measured values. Even at water activities as high as 0.75, where the viscosity is relatively low (∼250 Pa s), the S–E equation under-predicts water diffusion coefficients by 2–3 orders of magnitude. The observed significant breakdown of the S–E description thus emphasizes the need to make direct measurements of diffusion. While the S–E equation may be applicable for large molecules, it fails to predict water diffusion coefficients in SOM. Finally, we compare our water diffusion coefficient measurements with estimates produced from a model based on percolation theory,^30^ which assumed that the water diffusion coefficient in pure SOM is the same as that in pure amorphous sucrose. We have found water diffusion in α-pinene SOM to be faster than in sucrose solutions at the same water activity and temperature, possibly explaining some of the discrepancies between our measured data and the percolation theory estimate.

**Fig. 3:**
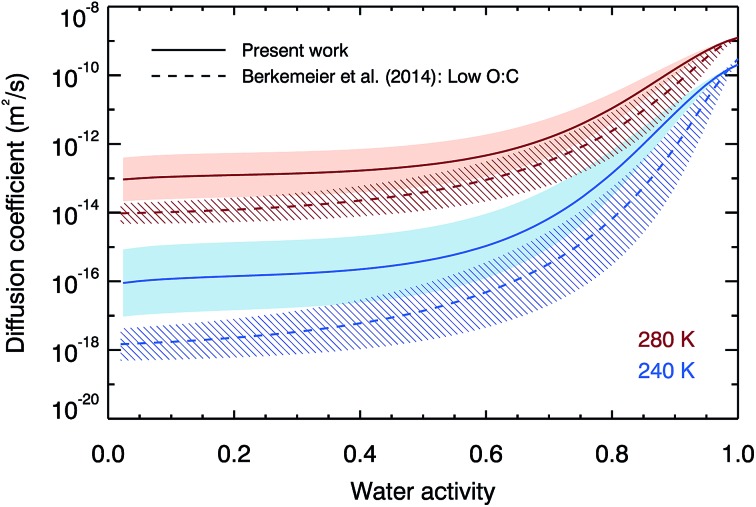
The fit to our experimental data (solid lines and shaded error regions) compared with the semi-empirical low oxidation predictions from Berkemeier, *et al.* ^53^ (dotted lines and hatched error regions) at 280 K and 240 K.

To further compare our laboratory data with the semi-empirical VFT-based approach of Berkemeier, *et al.*,^53^ Fig. 3 compares both the water activity and temperature variation of our water diffusion coefficient parameterisation with the semi-empirical predictions at 280 and 240 K. The shapes of the water activity dependence curves are similar, despite being described by equations of different forms. The temperature dependence is stronger in the Berkemeier, *et al.* ^53^ prediction which predicts smaller diffusion coefficients than are measured here, although our results are in agreement within error above a water activity of 0.4. At low water activities, our best estimates of diffusion are higher than those of Berkemeier, *et al.* ^53^ by one order of magnitude at 280 K, and two orders of magnitude at 240 K. In a situation where a droplet has a uniform water activity, temperature and diffusion coefficient, timescales for diffusion are inversely proportional to diffusion coefficient, and thus we predict timescales that are an order of magnitude faster at 280 K, and two orders of magnitude faster at 240 K.

### Timescales for hygroscopic growth and shrinkage

**Fig. 4:**
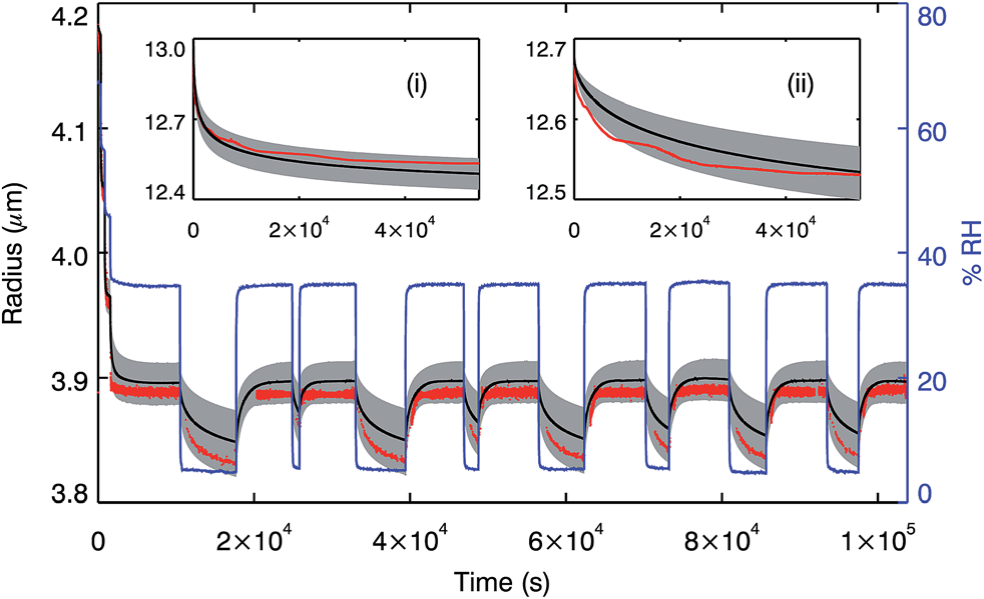
The main plot shows the changing radius of a sucrose droplet following a repeatedly stepped RH profile (blue line) at room temperature, measured using optical tweezers (red points). The same RH profile was run through the model for a droplet with 1000 shells; the output radius is shown by the black line, with the grey shaded region corresponding to the error in the measured RH of ±2%. Inset (i) shows the size change recorded in an EDB for a 13.03 μm droplet confined and equilibrated at 53% RH experiencing a rapid step change (halftime for RH change ≪ 1 s) to 20% RH (red line). The equivalent model output is shown in black, run with 3000 shells, with the grey region corresponding to a ±2% error in starting water activity and chamber RH. Similarly, inset (ii) shows the size change recorded in an EDB for a 12.68 μm droplet confined and equilibrated at 35% RH experiencing a rapid step change to 15% RH (red line). Again, the model output is shown in black, with the grey region corresponding to a ±2% error in starting water activity and chamber RH. Uncertainties in the experimentally determined radii are estimated to be ±50 nm.

To investigate water uptake and loss from aerosol particles in response to changing RH and temperature conditions, rather than the macroscale cylindrical disk used in our measurements for SOM, a multi-shell water diffusion model was developed similar to those described by Zobrist, *et al.* ^42^ and Lienhard, *et al.* ^43^ The model works by dividing a droplet into concentric shells whose concentration, and accordingly diffusion coefficient, varies as time progresses according to Fick's first law. To verify the accuracy of the model, we compared the output with laboratory measurements of changes in aqueous sucrose particle radii driven by RH changes, measured in both electrodynamic balance (EDB) and optical tweezers experiments. Using the same RH profiles as the laboratory experiments, the model simulated these changes in radii using our water diffusion coefficients measured in aqueous sucrose with the Raman tracer technique.^46^ Fig. 4 demonstrates that the model is able to reproduce the laboratory data within the accuracy of the experiments. We confirm that diffusion coefficients measured in a 200 μm disk can be used to simulate laboratory measurements on droplets of 13 μm and 4 μm radius, smaller in volume by a factor of <10^–4^, thus confirming the validity of our experimental and modelling approach. For further details see the ESI.†

In order to explore the response of SOM aerosol particles to changes in RH at 280 K, simulations of the time-resolved size and composition were performed for a 100 nm diameter water-soluble α-pinene SOM particle experiencing step changes in RH. The model was set up with 150 shells and used the SOM water diffusion coefficient parameterisation described above. It was used to calculate the timescales for hygroscopic growth by condensation and evaporation of the particle in response to different sized steps, up and down, in RH between 10 and 90% RH. For all steps tested, the time taken for the radius of the particle to increase or decrease by 95% of the total predicted size change was less than 0.01 s (see Fig. S6†).

It has been suggested that particle residence times in the dry and humid sections of a hygroscopic tandem differential mobility analyser (HTDMA; typically on the order of 10 s^59,60^) may not be sufficiently long to allow for complete equilibration of viscous aerosol water content with surrounding RH.^61^ This could lead to erroneous measurements of the hygroscopicity parameter, *κ*, for example due to an over-estimation of the dry diameter in the case of incomplete water loss due to “trapped” water inside a glassy shell. However, our model predictions show that, at 280 K, 100 nm α-pinene SOM particles will complete 95% of their total size change within 0.01 s for any step change in RH between 10 and 90%. Assuming that diffusion coefficients increase with increasing temperature, we find that water diffusion in our α-pinene SOM is sufficiently fast at room temperature that equilibration would be achieved in an HTDMA, in agreement with previous work.^62^ However, SOM from other sources may be considerably more viscous, with potential impacts on diffusion coefficients and therefore equilibration timescales.

**Fig. 5:**
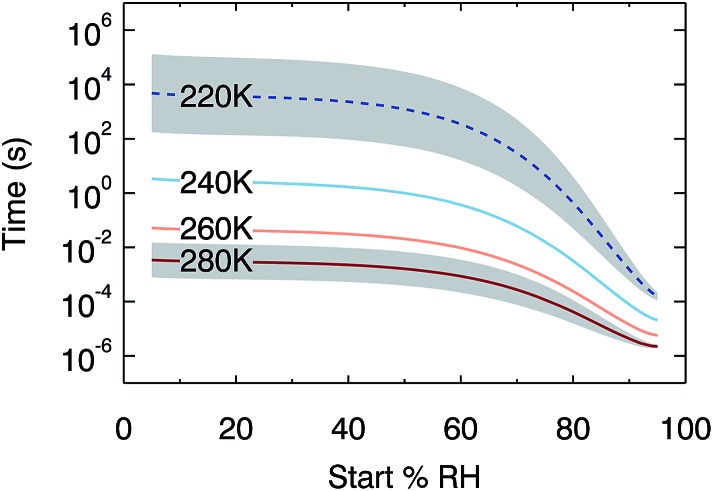
Modelled times for an increase in water activity of 0.01 at the centre of a 100 nm diameter particle following a 2% step up in RH. Below 240 K we use an extrapolation of our water diffusion coefficient fit, indicated by a dotted line. Timescales for equivalent steps down in RH are very similar. The grey shaded region indicates our estimated uncertainty (see ESI† for details of error analysis).

Although water diffusion is fast near room temperature, timescales for equilibration increase at lower temperatures. In order to quantify this kinetic limitation, Fig. 5 shows the output of 384 model runs where temperature is constant within a run and RH is increased by a step of 2%, over the temperature range from 220 K to 280 K. The plot shows the time taken for the water activity in the centre of a 100 nm droplet to increase by 0.01 (*i.e.* 50% of the change required to come back to equilibrium). At temperatures of 260 K and above, these timescales are faster than 1 s across the RH range 5% to 95%. At lower temperatures, however, slow diffusion kinetically limits the response in composition: at 240 K, the half-time for the water activity response at low RH is 3 s. We extrapolate diffusion coefficient dependence on temperature to estimate the further increase in these timescales at upper-tropospheric temperatures. Whilst such an extrapolation should be treated with caution, it strongly suggests that diffusion is so inhibited at 220 K that small changes in water activity may take hours. Moreover, larger organic species in SOM might be expected to diffuse even more slowly than water.^63^ Our results therefore imply that at low temperatures, equilibrium thermodynamic partitioning between condensed and gas phases may not be achieved, placing kinetic limitations on aerosol processing.

It is important to stress that timescales depend strongly on size, and increasing the starting diameter of particles in the model to 1 μm increases the timescales for diffusional mixing by a factor of 100. In our experiments, the 200 μm disk can take weeks to equilibrate with the surrounding water vapour, but a 100 nm aerosol particle of the same material under the same conditions will take less than a second to equilibrate.

### Modelling aerosol water uptake in an atmospheric updraft

**Fig. 6:**
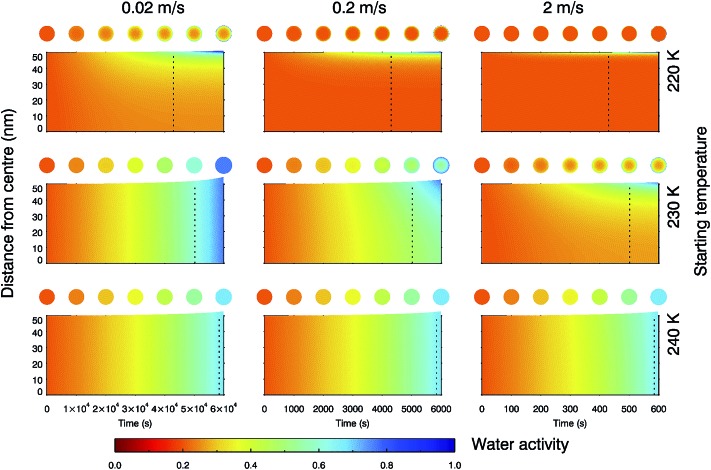
Water activity across droplet radius as a 100 nm particle follows an updraft of 0.02, 0.2 and 2 m s^–1^ (left to right) with temperature decreasing according to the dry adiabatic lapse rate and fixed water vapour partial pressure. Droplets start in equilibrium with their surrounding RH at a homogeneous water activity of 0.2, at a temperature of 220, 230 or 240 K (top to bottom). The resulting changes in RH and temperature with time are shown in ESI Fig. 7.† The dotted lines indicate the point at which the RH_ice_ increases above 100% and heterogeneous nucleation is feasible.

The multi-shell diffusion model was further configured to simulate the changing water activity of a 100 nm water-soluble α-pinene SOM particle as it was exposed to conditions equivalent to unsaturated air rising and cooling according to the dry adiabatic lapse rate (see Fig. S7†). Here we used an extrapolation of the water diffusion coefficient parameterisation for trajectories starting at 220, 230 and 240 K. Fig. 6 shows the changing water activity within the droplet for nine cases corresponding to three updraft velocities and three starting temperatures typical of synoptic cirrus formation. In each case the SOM particle starts at 20% RH, in equilibrium with the surrounding water vapour.

It can be seen that the faster the updraft velocity and the lower the temperature, the more marked the radial inhomogeneity in water content inside a SOM particle. A starting temperature of 230 K and updraft speed of 2 m s^–1^, or a starting temperature of 220 K and updraft speeds between 0.02 and 2 m s^–1^, may lead to situations in which the core of a particle remains at a low water activity when the environmental conditions are above 100% RH_ice_. This suggests that some fraction of the water soluble component of SOM may be present in a highly viscous or glassy phase under upper tropospheric conditions relevant to synoptic cirrus formation, and hence may have the capacity to nucleate ice as observed in the laboratory.^33,37–39^ For a starting temperature of 240 K, water diffusion coefficients are rapid enough for a SOM particle to maintain equilibrium with surrounding water vapour at updraft speeds between 0.02 and 2 m s^–1^. Simulations performed at temperatures above 240 K gave similar results, with droplets retaining their radial homogeneity in water activity. These liquid particles will have no amorphous solid core and therefore the mode of nucleation would change. A liquid solution droplet with no ice nucleating particles would be expected to freeze homogeneously according to the water activity criterion.^64^

Fig. 6 demonstrates that SOM particle water content (and consequently phase) can be non-uniform under certain conditions, and depends on the temperature and RH history of the particle. This sort of radial inhomogeneity was directly observed by Bones, *et al.* ^61^ in sucrose at room temperature, and was proposed to exist in SOM by Berkemeier, *et al.*,^53^ who used estimates of diffusion coefficients to show that kinetic limitations to water diffusion may create core–shell morphologies potentially favourable for ice nucleation. In order to evaluate the impacts of SOM particles in heterogeneous and multiphase chemistry, and thus its impact on tropospheric composition, it is necessary to have a clear understanding of whether particles are present in the form of a liquid, solid or a combination of both.^65,66^ The possible presence of a highly viscous or glass phase should be borne in mind in future laboratory studies examining the chemical processing in the presence of SOM.

In this work, laboratory-generated SOM is used, which has some known differences to atmospheric SOM: it is typically less oxidised and more volatile than ambient particles.^67^ Higher oxygen to carbon ratio in α-pinene SOM correlates with higher *T*_g_,^53^ indicating that atmospheric SOM may be more viscous than the SOM studied here. Recently, O'Brien, *et al.* ^68^ found that viscosity and/or surface tension could be higher in ambient organic particles than laboratory-generated SOM, attributing this to variations in chemical aging time and the complexity of field aerosol. Assuming that higher viscosity is associated with slower diffusion, this implies that the diffusion coefficients of water in atmospheric SOM might be lower than those we measured in our laboratory study. On the other hand, the duration of our experiments may lead to the unavoidable evaporation of some semi-volatile components of SOM, which may have the effect of decreasing the measured diffusion coefficients. We used SOM that was generated at low RH, from a single precursor, and this cannot necessarily be assumed to be characteristic of real atmospheric SOM. It should be emphasized that we have measured the diffusion of water, a highly mobile component, but the diffusion of larger organic molecules in SOM is much slower.^16,63^ This may lead to inhibition of condensed-phase chemistry in situations where water diffusion is unimpeded and cause a kinetic limitation to gas-particle partitioning of semi-volatile organic compounds. Future work should therefore focus on measuring the diffusion of larger molecules in SOM.

This study highlights the importance of directly measuring diffusion in order to determine how molecules are transported in SOM. We have shown that water diffusion is not kinetically limited in the water-soluble component of α-pinene SOM at 280 K, but slows dramatically as temperatures decrease. Under conditions relevant to the upper troposphere, radial variations in phase develop which may have important consequences for aerosol chemistry and ice nucleation. The role of slow diffusion in SOM needs to be explored further in order to quantify its impact on atmospheric chemistry and clouds, which may in turn affect climate.
