# Supplementary material for: Water diffusion in atmospherically relevant α-pinene secondary organic material
Source: Chem Sci. 2015 Jun 4;6(8):4876–83. doi: 10.1039/c5sc00685f (PMC5502394; doi:10.1039/c5sc00685f)
Supplement: Supplementary file 1 [file SC-006-C5SC00685F-s001.pdf]

Electronic Supplementary Material (ESI) for Chemical Science.
This journal is © The Royal Society of Chemistry 2015

Supplementary Information

("Water diffusion in atmospherically relevant α-pinene secondary organic material", Price et al.)

Production of secondary organic material for diffusion measurements.

The particle population of α-pinene secondary organic materials (SOM) was generated in a flow tube reactor[1]. The flow tube was made of glass and had an inner diameter of 48.2 mm and a length of 1.30 m. It was operated in a laminar-flow regime (Reynolds number of 9.4±0.5) for a residence time of 38±1 s. The flow tube was equipped with ozone (Ecosensors, UV-100) and temperature sensors (National Instrument, USB-TC01) at the inlet. The flow tube reactor was housed in an external temperature-controlled, double-hulled, water-jacketed housing that contained both the flow tube and the associated gas flow system. The temperature was 20.0±0.1 °C during each experiment and accurate temperature control was critical for reproducing experimental results of particle number-diameter distributions. The total pressure inside the flow tube, measured using a barometer (Omega PX409), ranged from 1.00 to 1.01 atm. An air flow of 3.0 sLpm from an Aadco 737 Pure Air Generator passed through an ozone generator (Jelight, Model 600), producing 12 to 16 ppm of ozone. A syringe injector (CHEMYX, Fusion Touch 200 Model) provided (+)-α-pinene (Sigma-Aldrich, ≥ 99% purity, 97% enantiomeric excess) and 2-butanol (Sigma-Aldrich, ≥ 99.5% purity; used as OH scavenger) at a dilution ratio of 1:49. The solution from the injector was introduced into the flow tube with an additional flow of pure, dry air at 0.50 sLpm. The concentration of α-pinene in the total flow was set to be 6 ppm by adjusting the syringe injection rate. The concentrations reported here are those at the inlet of the flow tube. The aerosol particles flow out of the flow tube was measured by scanning mobility particle sizer (SMPS), given a mass concentration of 10 mg m^{-3}. The particles were also collected onto a quartz filter for 48 to 63 hours. The SOM on the filter was then extracted by 5.00 mL of ultra-pure water through ultrasonication and used for this experiment. As noted by Renbaum-Wolff, et al. [2], a significant amount of total particle mass can be extracted from filters using water[3], and the use of 50:50 methanol:water instead of water for the extraction of similar materials has been shown to make little difference to the extract composition[4].

Raman spectral fitting procedure for α-pinene secondary organic material

In order to determine the diffusion coefficient of water, D_{water}, in secondary organic material (SOM), we extracted the relative intensities of O-H and O-D bands from the Raman spectrum at various points on the sample. The O-H and O-D stretch bands in the SOM+H_2O and SOM+D_2O solutions were relatively small in comparison with the C-H stretch bands and the background signal. In addition, the background signal did not remain constant throughout all experiments, for example due to condensation from the lab on the outside of the glass window to the RH control chamber at low stage temperatures. For this reason, the spectral fitting procedure had to be carefully constrained, with certain parameters allowed to vary and others held fixed. We emphasize that the fits produced here are for the purpose of quantifying the relative integrated band intensities of the O-H and O-D bands; they are not intended as accurate band assignments.

In order to constrain the C-H peak fitting, a spectrum was taken of the background (i.e. the hydrophobic glass side without sample). A Gaussian curve plus a constant was fitted to this, as shown in Fig. S1. The width and wavenumber of this Gaussian were held fixed in all subsequent fits, with the intensity allowed to vary. The constant was determined by the minimum intensity recorded in each spectrum: it had to be between 80% and 100% of this minimum value. To fit a set of peaks to the C-H stretch region alone, the O-H and O-D bands needed to be removed from the spectrum. To do this, a droplet of SOM solution was exposed to H_2O vapour at 70% RH until it reached equilibrium with the surrounding vapour phase, and a high-quality spectrum was taken. The droplet was then exposed to D_2O vapour until complete exchange had taken place and no O-H peaks remained. Again, a high-quality spectrum was taken. A background fit (using the constrained Gaussian plus constant described above) was performed using the upper and lower ends of each spectrum, then subtracted. The spectra were then normalised to the peak intensity; the results of this are shown in Fig. S2. To obtain a “spectrum” without either O-H or O-D bands, the lower half of the SOM+H_2O spectrum was stitched to the upper half of the SOM+D_2O spectrum. 7 Gaussian curves were fitted to the remaining C-H stretch, along with a shallow, broad Gaussian that was required to account for a slightly raised background, as shown in Fig. S3. The relative peak positions and intensities of the 7 C-H Gaussians were fixed in all subsequent fits. The peak position of the broad, shallow Gaussian was fixed relative to the 7 C-H Gaussians, but its intensity and width was allowed to vary.

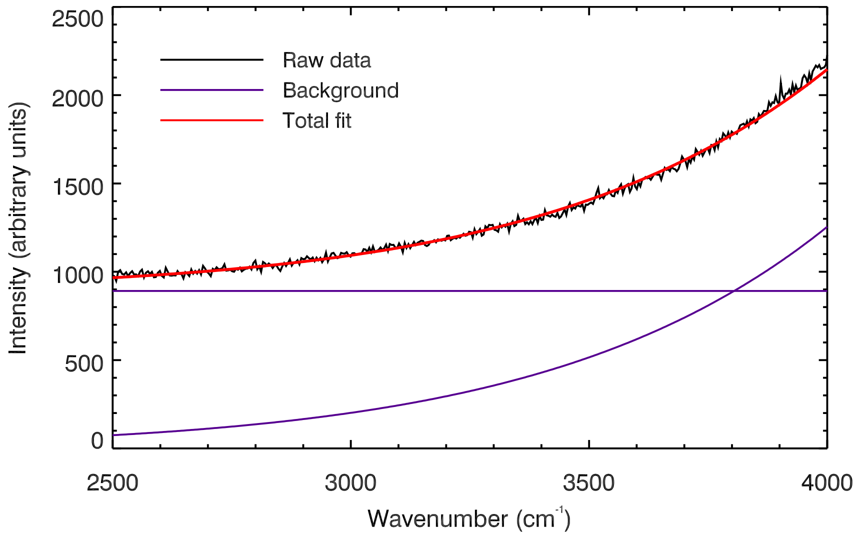

Figure S1. The Raman spectrum of a hydrophobic glass slide. The purple lines show a Gaussian curve and a constant which add to give the red fit to the background.

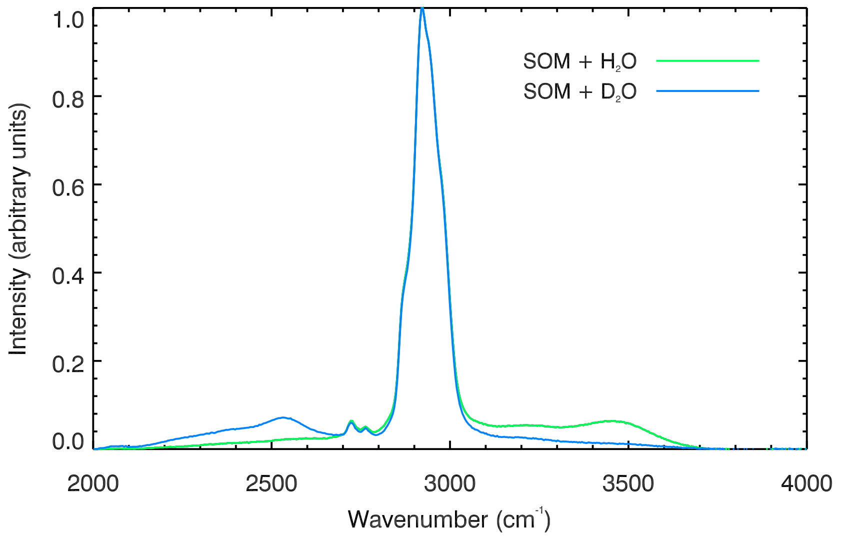

Figure S2. Background corrected, normalised spectra of aqueous α-pinene SOM after long periods of exposure to H_2O (green) and D_2O vapour (blue) at 70% RH.

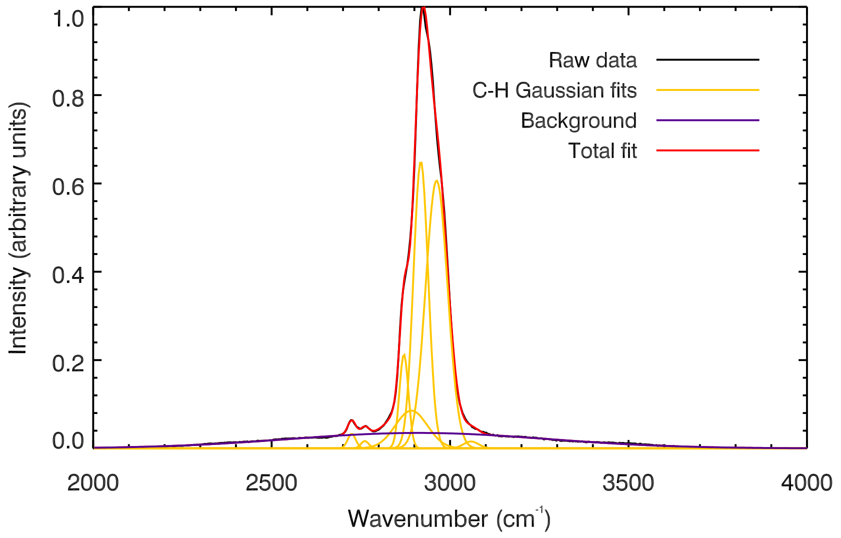

Figure S3. The Raman spectrum of aqueous α-pinene SOM with O-H and O-D peaks removed. Gaussian fits to the C-H stretch band (yellow) and the background (purple) shown here are used to constrain the fitting routine. The total fit (red) overlays the raw data (black) to the point that it is barely visible.

Figure S4 shows the total fit (red) to three spectra of varying H_2O/D_2O concentrations, using the constraints described. When fitting to a spectrum taken during a H_2O/D_2O exchange experiment, three variable-intensity Gaussians representing the O-D stretch band were constrained to lie within the wavenumber range 3200 to 3500 cm^{-1}, and had FWHMs between 45 and 235 cm^{-1} (green). Another three variable-intensity Gaussians representing the O-H stretch band were constrained to lie within the wavenumber range 2300 to 2600 cm^{-1}, and also had FWHMs between 45 and 235 cm^{-1} (blue).

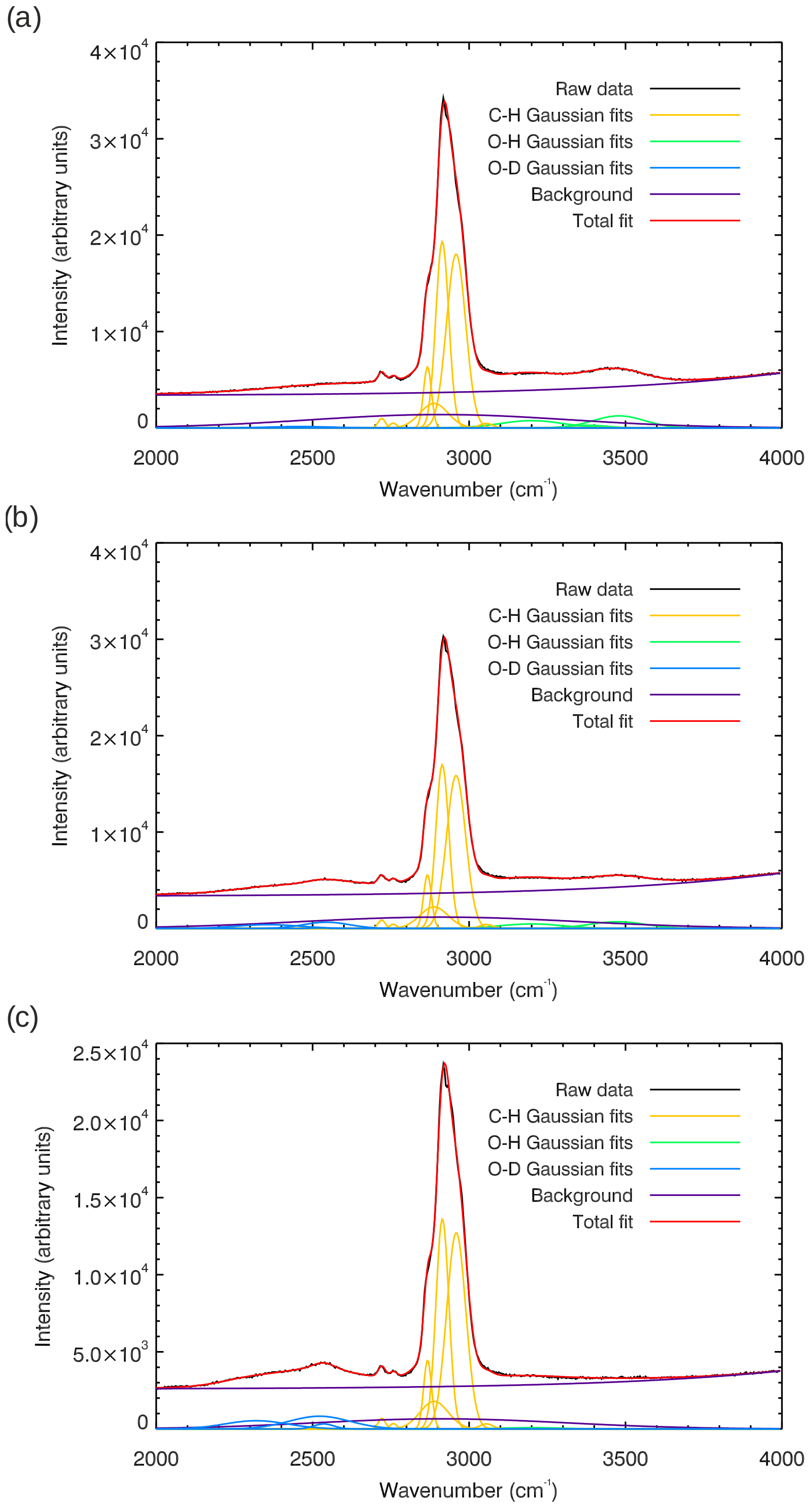

Figure S4. Results of the full fitting procedure for aqueous SOM containing (a) mostly H_2O, (b) similar amounts of H_2O and D_2O, and (c) mostly D_2O.

For each spectrum obtained, the fitting procedure was performed three times: firstly with all Gaussian peaks described above available to the fitting routing, secondly without the three O-H

Gaussians available, and thirdly without the three O-D Gaussians available. This allowed an f-test to be performed using the χ^2 values for the different fits, so that the level of significance of the O-H and O-D bands could be determined. Spectra where the probability that the decrease in χ^2 associated with the addition of either the O-H or O-D bands was due to chance alone was greater than 0.01 were excluded from subsequent analysis in the diffusion coefficient surface fit.

Determining the water diffusion coefficient from $\boldsymbol{\varphi(r,t)}$

The solution to Fick's second law used to find the diffusion coefficient in a disk of radius a whose edge is maintained at concentration φ_0 is

$$\varphi(r,t) = \varphi_0 + 2(\varphi_1 - \varphi_0)\sum_\alpha \frac{1}{\alpha J_1(\alpha)} e^{\frac{-\alpha^2 Dt}{a^2}} J_0\left(\frac{\alpha r}{a}\right) \qquad (1)$$

where φ_1 is the concentration at $t = 0$ everywhere in the disk except at the edge ($r = a$) at $t = 0$, J_0 and J_1 are Bessel's functions of order 0 and 1, respectively, and α are the positive roots of the equation $J_0(x) = 0$ [5]. The results of the spectral fitting procedure were used with the above equation, with φ as the O-D bond concentration, to find the diffusion coefficient of D_2O in the sample. Because the total amount of water (D_2O plus H_2O) and solute remained constant throughout the experiment, φ was written as the fractional concentration of O-D bonds relative to the total O-D and O-H bonds at each spectral location and time:

$$\varphi = \frac{A_{OD}}{A_{OD} + \frac{1}{\sqrt{2}} A_{OH}} \qquad (2)$$

where $A_{OD}$is the integrated intensity of the O-D stretch and A_{OH} is the integrated intensity of the O-H stretch.

In our previous work, $\varphi_0$was set to 1 (the fractional O-D concentration maintained at the edge of the disk) and φ_1 set to 0 (the O-D concentration everywhere except the edge at $t = 0$). To determine the D_{water} at a given RH, a surface fit of Eq. (1) with the diffusion coefficient as the sole variable parameter was performed to the measured values of $\varphi(r,t)$. Due to the difficult nature of the fitting procedure in SOM (caused by shallow peaks and large background signals), the fit could sometimes be improved by allowing φ_0 and φ_1 to vary, along with D_{water}. The effect of allowing φ_0 and φ_1 to vary did not appear to systematically alter the values of D_{water} that were determined, and in most cases the fitting routine found the best fit values of φ_0 and φ_1 to be 1 and 0, respectively. It did, however, improve the quality of the surface fit in the few cases where the large background signal was particularly large. An example of the fitted surface is shown in Fig. S5, with the experimentally determined φ values are connected to the fitted surface by black lines.

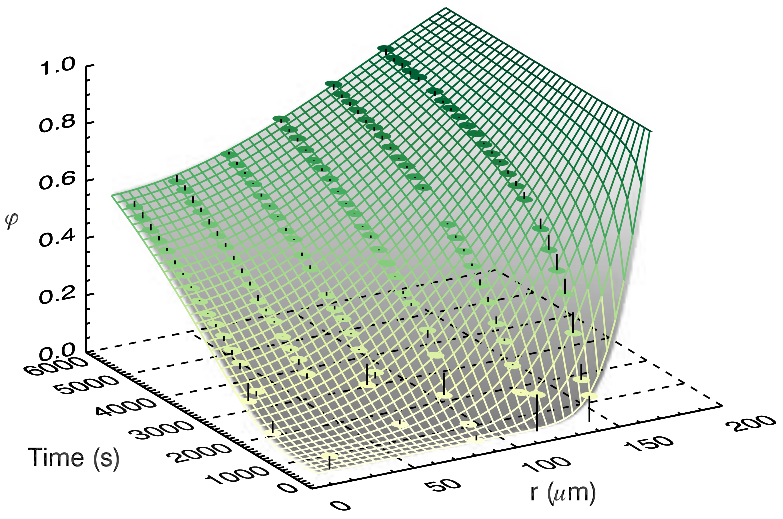

Figure S5. An example of the surface fit to $\varphi(r, t)$ used to determine the diffusion coefficient of water, where experimentally determined values of φ are joined to the fitted surface by black lines.

The number of spectra collected for each experiment depended on the duration of each experiment, which was determined by the rate of diffusion at each relative humidity and temperature. Experiments at high diffusion coefficients resulted in the collection of several hundred spectra; experiments at low diffusion coefficients generated several thousand spectra.

Parameterisation of the diffusion coefficient of water in SOM

The water diffusion coefficient data was parameterised using an empirical fit to a Vignes-type equation[6-8]:

$$D_{water} = (D^0_{water})^{x_w\alpha}\left(D^0_{SOM}\right)^{1-x_w\alpha} \qquad (3)$$

where D^0_{water} is the temperature-dependent self-diffusion coefficient of water[9] and D^0_{SOM} is the diffusion coefficient of water in amorphous SOM at water activity, $a_w = 0$. D^0_{SOM} is expressed in the form of a Vogel-Fulcher-Tammann (VFT) relationship[10]:

$$\log_{10}\left(D^0_{SOM}/\mathrm{m^2 s^{-1}}\right) = -\left(A + \frac{B}{T - T_0}\right) \qquad (4)$$

in which A, B and T_0 are fitted parameters indicating the high temperature limit of the diffusion coefficient, the fragility and the temperature at which the diffusion coefficient would diverge, respectively. x_w is the mole fraction of water, calculated from the water activity using the effective hygroscopicity parameter (κ_{org})[11, 12]:

$$x_w = \left[\frac{M_w}{M_{org}}\frac{\rho_{org}}{\rho_w}\frac{(1-a_w)}{\kappa_{org}a_w} + 1\right]^{-1} \qquad (5)$$

where M_w is the molar mass of water, ρ_w is the density of water, M_{org} is the effective molar mass of SOM, ρ_{org} is the density of SOM. We use a value of 180 g·mol^{-1} for the effective molar mass of SOM[13, 14], and an effective density of SOM of 1.2 g·cm^{-3}[15]. For κ_{org}, we use a value of 0.1, calculated using the measured O:C ratio for SOM from the flow tube of 0.38±0.01[15-17], according to the linear relationship given by Lambe, et al. [18]. This κ_{org} value is consistent with previous measurements[11, 19-22]. α is an activity coefficient[6, 23]:

$$\ln\alpha = (1 - x_w)^2[C + 3D - 4D(1 - x_w)] \qquad (6)$$

C and D are linear in temperature below a certain temperature, T_{cutoff}, and constant at higher temperatures, such that $C = p + qT_\alpha$ and $D = r + sT_\alpha$, where $T_\alpha = \begin{cases} T, & T < T_{cutoff} \\ T_{cutoff}, & T \geq T_{cutoff} \end{cases}$, with p, q, r, s and T_{cutoff} as fitted parameters. The fit was performed using the Levenberg-Marquardt technique[24]; the best fit parameters, together with their estimated errors, are shown in Table S1.

	p	q	r	s	T_{cutoff} (K)	A	B	T_0 (K)
**Best fit value**	-13	0.043	-10.5	0.035	230	7.4	650	165
**Estimated error**	1	0.006	0.7	0.003	3	0.3	60	4

Table. S1. Parameters for the Vignes-type fit described in Equations 3-6.

Determining errors in the fitted parameters of $D_{water}(a_w, T)$

The errors in the individual diffusion coefficient measurements are calculated in the same way as in our previous work [25]. The upper error is calculated using the upper limit on the disk's radius, the upper limit on the time taken for the switch from H_2O to D_2O vapour, and the random error in the surface fit due to errors in φ. Likewise, the lower error is calculated using the lower limit on the disk's radius, the lower limit on the time taken for the switch from H_2O to D_2O vapour, and the random error in the surface fit due to errors in φ.

The error in the fitted $D_{water}(a_w, T)$ comes from not only the error in the diffusion coefficients, but also the error in RH and temperature. Due to the complicated nature of these errors, the uncertainty in the fitted diffusion coefficient was estimated by performing the Vignes-type equation fit 100,000 times. Each time, the RH and temperature to be used in the fit for each of the 26 datapoints was drawn from a probability distribution function. For the temperature errors, a normal distrubition was used, with a standard deviation corresponding to the standard deviation in the continuous temperature measurement (as measured using a calibrated PT100 temperature sensor embedded in the cooled metal block on which the sample was placed). The RH errors had to take into account errors due to potential radial inhomogeneities, errors due to the difference in vapour pressures between H_2O and D_2O, and the standard deviation in the continuous RH measurement by the dewpoint hygrometer. To do this, a skew normal distribution was assigned to each measurement, with the mode corresponding to the best estimate of RH and the 5% and 95% limits corresponding

to the estimated lower and upper bounds. The reported errors in the fitted parameters are the χ^2-weighted standard deviations in the output from these 100,000 fits.

Viscosity measurements on flow tube-generated SOM

SOM was produced in a flow tube using an α-pinene concentration of 5 ppm and ozone concentration of 12 ppm, and collected over 11-14 hours on Teflon filters before extraction with water. The mass concentration at the flow tube outlet was 6 x 10^3 µg m^{-3}. The approach of using a poke-flow technique and subsequent simulations of fluid flow to determine limits of viscosity is described by Renbaum-Wolff, et al. [2], with the sole difference here being that simulations of material flow were used to determine the lower limit of viscosity (as opposed to lower limits derived through the use of lower limits of viscosity being inferred from use of the bead-mobility technique at high RH, as was done previously). For simulations of the lower limit of viscosity, a slip length of 5 nm, a surface tension of 32 mN•m^{-1} were used. Contact angles were 63° at 40 % RH and 70° at 30 % RH, whilst for simulations of the upper limit of viscosity at 40 % RH, a slip length of 10 um, a surface tension of 75 mN•m^{-1}, and a contact angle of 60° were used.

Timescales for hygroscopic growth and shrinkage at 280 K for varying step changes in RH

Figure S6 shows the timescales for hygroscopic growth (upper left side of plots) and shrinkage (lower right side of plots) of the particle in response to changes from various starting RHs (x-axes, corresponding to the homogeneous starting $a_w \times 100$) to final RHs (y-axes). The time taken for the radius of the particle to increase/decrease by (a) 50% and (b) 95% of the total predicted size change is represented by the colour of each datapoint. It can be seen that water uptake and loss occur on timescales much faster than one second in these small particles, even at low RH, even though similar materials are known to have high viscosities and super-micron particles are known to shatter when subjected to mechanical force[2].

The first 50% of change in size occurs most rapidly in high-a_w particles when they are exposed to a sudden large decrease in RH (bottom right of Fig. S6(a)), whereas low- a_w particles exposed to high RH are comparatively slow to take up water (top left of Fig. S6(a)). Water can be lost rapidly from a more dilute droplet, whereas water uptake to a highly concentrated droplet is initially inhibited by low diffusion coefficients and accelerates only when enough hygroscopic growth has occurred to soften the outer layers of the particle. Low- a_w droplets experiencing small changes in RH are the slowest to complete 95% of their size change (bottom left of Fig. S6(b)), although these are conditions where the change in composition and size are minimal.

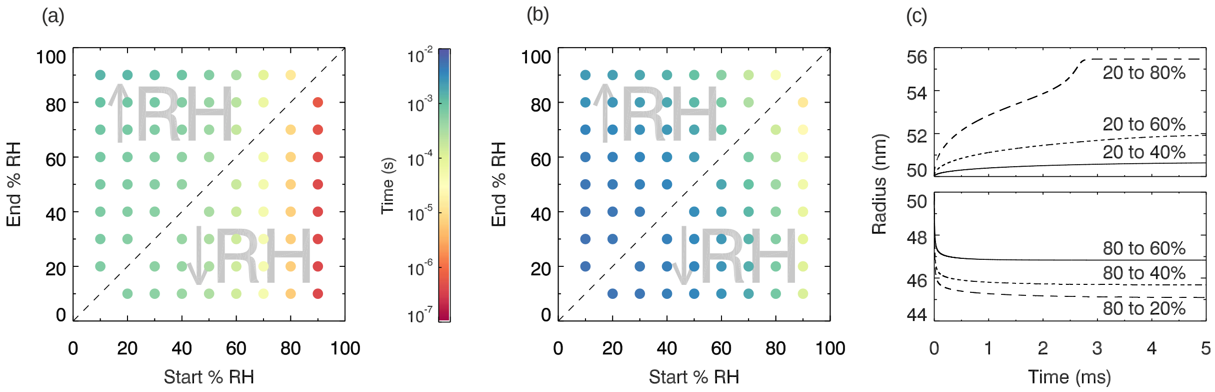

Figure S6. Hygroscopic growth and shrinkage times for an α-pinene SOM droplet of 100 nm starting diameter following various step changes in RH at 280 K. (a) Time for the radius to change by 50% of the total amount predicted for each RH step. (b) Time for the radius to change by 95% of the total amount predicted for each RH step. (c) Examples of size changes with time.

Water diffusion model

The water diffusion model tracks water diffusion into and out of a spherical aqueous droplet in response to changing RH and temperature conditions, in a similar way to that described by Zobrist, et al. [26] and Lienhard, et al. [6]. The model is used for both sucrose aqueous solutions (for the purpose of model comparison with laboratory data) and α-pinene SOM. The droplet is split into a number of concentric shells, whose concentration and diffusion coefficient are allowed to vary as time progresses according to the flux between shells calculated using Fick's first law. The model assumes that the outer shell always has a water activity corresponding to the surrounding relative humidity, with the model following a RH and temperature profile input by the user. The RH and temperature to be used in each timestep are calculated by linearly interpolating between the two nearest points in the user-defined environmental profile.

The maximum and minimum percentage change allowed in the number of water molecules in the shell which sees the biggest change in the number of water molecules in a given timestep is restricted; after sensitivity testing, maximum and minimum percentage changes of 1 and 2%, respectively, were found to be appropriate in most cases. These were reduced to 0.005 and 0.01% when the model was required to resolve steep concentration gradients (for each scenario used in the model, convergence checks were performed to ensure that the number of shells and the minimum and maximum percentage changes did not affect the result). For sucrose at room temperature, the solute mass fraction is related to water activity using the relationship given in Zobrist, et al. [26], and for the α-pinene SOM, the hygroscopicity parameter, κ_{org} = 0.10, is used to relate water activity to mole fraction [12]. The diffusion coefficient of water in each shell is calculated based on its water activity and temperature (for sucrose using the parameterisation given in Price, et al. [25] at 296.5 K and for α-pinene SOM equation 1 from the main text is used).

Output from the model was compared with laboratory data for sucrose water uptake and loss performed using aerosol optical tweezers and an electrodynamic balance (EDB), the experimental method for which has been described in detail in previous publications[27-31].

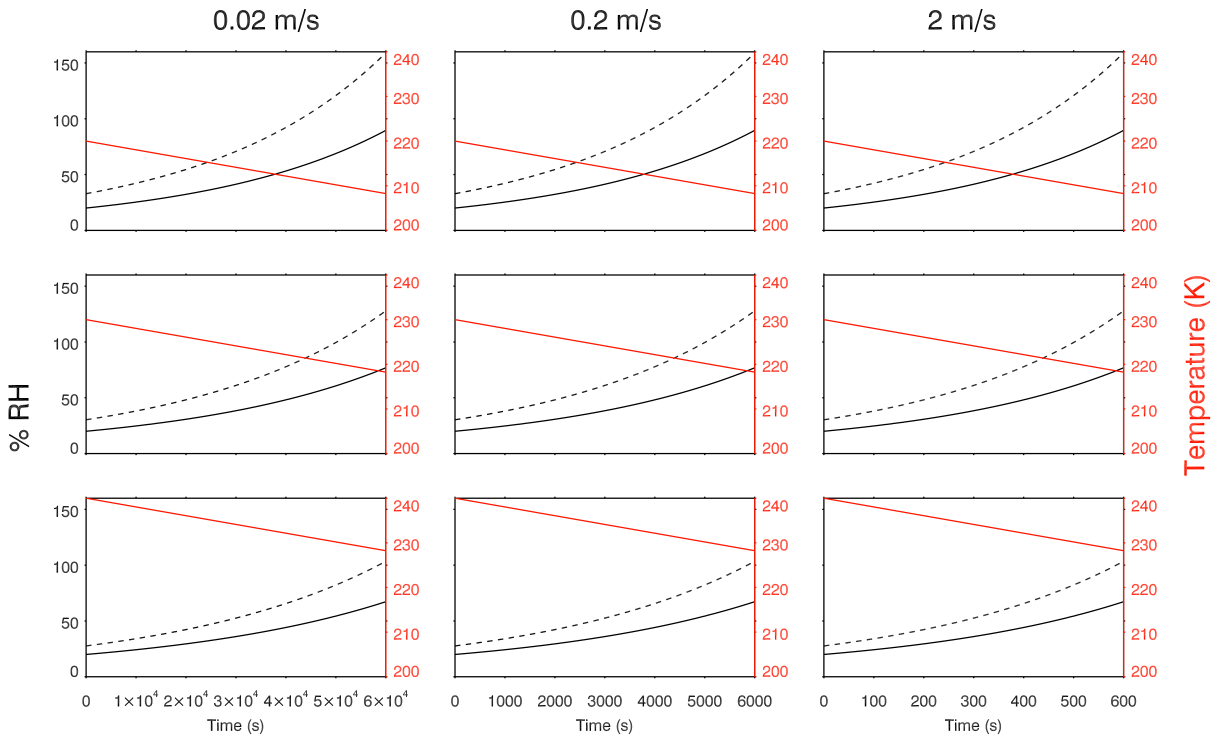

Figure S7. The relative humidity (black solid and dotted lines, showing RH_{liq} and RH_{ice}, respectively) and temperature (red lines) profiles used in the model simulation of aerosol water uptake in an atmospheric updraft. RH was calculated according to Murphy and Koop [32].

T (K, ± 0.5)	Water activity	Measured D_{water} (m^2/s)	D_{water} parameterisation (m^2/s)
279.9	$0.27^{+0.02}_{-0.03}$	$1.8^{+0.5}_{-0.6} \times 10^{-13}$	$1.3^{+4.4}_{-1.0} \times 10^{-13}$
279.9	$0.74^{+0.03}_{-0.03}$	$4.0^{+0.3}_{-0.3} \times 10^{-12}$	$3.2^{+7.4}_{-2.2} \times 10^{-12}$
279.8	$0.69^{+0.02}_{-0.04}$	$2.4^{+0.4}_{-0.1} \times 10^{-12}$	$1.3^{+3.5}_{-0.9} \times 10^{-12}$
279.7	$0.38^{+0.02}_{-0.04}$	$3.6^{+1.7}_{-1.3} \times 10^{-13}$	$1.5^{+5.1}_{-1.2} \times 10^{-13}$
279.7	$0.78^{+0.02}_{-0.03}$	$4.0^{+0.5}_{-0.2} \times 10^{-12}$	$7.0^{+13.6}_{-4.6} \times 10^{-12}$
279.7	$0.77^{+0.02}_{-0.03}$	$5.4^{+0.1}_{-0.1} \times 10^{-12}$	$6.4^{+12.7}_{-4.3} \times 10^{-12}$
279.6	$0.18^{+0.02}_{-0.02}$	$1.6^{+0.9}_{-0.7} \times 10^{-13}$	$1.1^{+4.0}_{-0.9} \times 10^{-13}$
279.6	$0.50^{+0.02}_{-0.05}$	$7.0^{+0.6}_{-0.5} \times 10^{-13}$	$2.3^{+7.4}_{-1.8} \times 10^{-13}$
279.6	$0.59^{+0.03}_{-0.06}$	$8.8^{+3.2}_{-0.3} \times 10^{-13}$	$4.0^{+12.5}_{-3.1} \times 10^{-13}$
279.6	$0.64^{+0.03}_{-0.04}$	$1.7^{+0.9}_{-0.4} \times 10^{-12}$	$6.7^{+19.8}_{-5.0} \times 10^{-13}$
279.6	$0.75^{+0.02}_{-0.03}$	$8.0^{+1.2}_{-0.4} \times 10^{-12}$	$3.4^{+7.7}_{-2.4} \times 10^{-12}$
279.4	$0.76^{+0.02}_{-0.03}$	$3.7^{+0.5}_{-0.3} \times 10^{-12}$	$4.0^{+8.8}_{-2.7} \times 10^{-12}$
279.4	$0.75^{+0.02}_{-0.03}$	$3.0^{+0.5}_{-0.3} \times 10^{-12}$	$3.3^{+7.6}_{-2.3} \times 10^{-12}$
278.9	$0.14^{+0.01}_{-0.01}$	$7.1^{+0.9}_{-0.8} \times 10^{-14}$	$1.0^{+4.0}_{-0.8} \times 10^{-13}$
274.2	$0.24^{+0.02}_{-0.02}$	$4.7^{+0.2}_{-0.3} \times 10^{-14}$	$6.6^{+24.3}_{-5.2} \times 10^{-14}$
273.5	$0.73^{+0.02}_{-0.06}$	$2.3^{+0.2}_{-0.1} \times 10^{-12}$	$1.4^{+3.8}_{-1.0} \times 10^{-12}$
273.3	$0.50^{+0.02}_{-0.04}$	$5.2^{+1.4}_{-1.2} \times 10^{-13}$	$1.1^{+4.0}_{-0.8} \times 10^{-13}$
263.1	$0.75^{+0.03}_{-0.05}$	$1.4^{+0.3}_{-0.1} \times 10^{-12}$	$8.1^{+23.3}_{-6.0} \times 10^{-13}$
263.0	$0.70^{+0.02}_{-0.04}$	$1.1^{+0.2}_{-0.1} \times 10^{-12}$	$2.8^{+9.8}_{-2.2} \times 10^{-13}$
262.9	$0.50^{+0.02}_{-0.03}$	$1.8^{+1.0}_{-0.8} \times 10^{-13}$	$2.8^{+12.1}_{-2.3} \times 10^{-14}$
262.6	$0.28^{+0.02}_{-0.02}$	$2.0^{+0.6}_{-0.4} \times 10^{-14}$	$1.4^{+6.4}_{-1.2} \times 10^{-14}$
253.7	$0.77^{+0.02}_{-0.06}$	$2.8^{+0.6}_{-0.2} \times 10^{-13}$	$4.0^{+12.8}_{-3.0} \times 10^{-13}$
252.9	$0.61^{+0.03}_{-0.10}$	$2.7^{+1.0}_{-0.8} \times 10^{-14}$	$1.5^{+7.6}_{-1.2} \times 10^{-14}$
252.0	$0.26^{+0.02}_{-0.05}$	$1.6^{+0.1}_{-0.2} \times 10^{-15}$	$2.3^{+13.2}_{-1.9} \times 10^{-15}$
243.8	$0.77^{+0.04}_{-0.10}$	$2.1^{+0.7}_{-0.3} \times 10^{-14}$	$8.3^{+34.9}_{-6.7} \times 10^{-14}$
243.5	$0.52^{+0.03}_{-0.10}$	$4.2^{+0.7}_{-0.7} \times 10^{-15}$	$1.1^{+7.5}_{-0.9} \times 10^{-15}$

Table S2. Water diffusion coefficients in α-pinene SOM measured using the Raman isotope tracer method. The final column gives the value and uncertainty in the Vignes-type parameterisation that corresponds to each measured data point.

References

1. M. Shrestha, Y. Zhang, C. J. Ebben, S. T. Martin and F. M. Geiger, *The Journal of Physical Chemistry A*, 2013, **117**, 8427-8436.
2. L. Renbaum-Wolff, J. W. Grayson, A. P. Bateman, M. Kuwata, M. Sellier, B. J. Murray, J. E. Shilling, S. T. Martin and A. K. Bertram, *Proceedings of the National Academy of Sciences*, 2013, **110**, 8014–8019.
3. W. A. Hall and M. V. Johnston, *Aerosol Science and Technology*, 2011, **45**, 37-45.
4. K. J. Heaton, R. L. Sleighter, P. G. Hatcher, W. A. Hall Iv and M. V. Johnston, *Environmental Science & Technology*, 2009, **43**, 7797-7802.
5. F. Bowman, *Introduction to Bessel Functions*, Dover Publications, New York, 1958.
6. D. M. Lienhard, A. J. Huisman, D. L. Bones, Y.-F. Te, B. P. Luo, U. K. Krieger and J. P. Reid, *Physical Chemistry Chemical Physics*, 2014, **16**, 16677-16683.
7. A. Vignes, *Industrial & Engineering Chemistry Fundamentals*, 1966, **5**, 189-199.
8. J. Matthiesen, R. S. Smith and B. D. Kay, *The Journal of Physical Chemistry Letters*, 2011, **2**, 557-561.
9. R. S. Smith and B. D. Kay, *Nature*, 1999, **398**, 788-791.
10. C. A. Angell, *Science*, 1995, **267**, 1924-1935.
11. M. D. Petters and S. M. Kreidenweis, *Atmos. Chem. Phys.*, 2007, **7**, 1961-1971.
12. T. Koop, J. Bookhold, M. Shiraiwa and U. Poschl, *Physical Chemistry Chemical Physics*, 2011, **13**, 19238-19255.
13. G. J. Engelhart, A. Asa-Awuku, A. Nenes and S. N. Pandis, *Atmos. Chem. Phys.*, 2008, **8**, 3937-3949.
14. S. M. King, T. Rosenoern, J. E. Shilling, Q. Chen and S. T. Martin, *Geophysical Research Letters*, 2007, **34**, L24806.
15. Y. Zhang, M. S. Sanchez, C. Douet, Y. Wang, A. P. Bateman, Z. Gong, M. Kuwata, L. Renbaum-Wolff, B. B. Sato, P. F. Liu, A. K. Bertram, F. M. Geiger and S. T. Martin, *Atmos. Chem. Phys. Discuss.*, 2015, **15**, 6821-6850.
16. A. C. Aiken, P. F. DeCarlo and J. L. Jimenez, *Analytical Chemistry*, 2007, **79**, 8350-8358.
17. A. C. Aiken, P. F. DeCarlo, J. H. Kroll, D. R. Worsnop, J. A. Huffman, K. S. Docherty, I. M. Ulbrich, C. Mohr, J. R. Kimmel, D. Sueper, Y. Sun, Q. Zhang, A. Trimborn, M. Northway, P. J. Ziemann, M. R. Canagaratna, T. B. Onasch, M. R. Alfarra, A. S. H. Prevot, J. Dommen, J. Duplissy, A. Metzger, U. Baltensperger and J. L. Jimenez, *Environmental Science & Technology*, 2008, **42**, 4478-4485.
18. A. T. Lambe, T. B. Onasch, P. Massoli, D. R. Croasdale, J. P. Wright, A. T. Ahern, L. R. Williams, D. R. Worsnop, W. H. Brune and P. Davidovits, *Atmos. Chem. Phys.*, 2011, **11**, 8913-8928.
19. Z. Juranyi, M. Gysel, J. Duplissy, E. Weingartner, T. Tritscher, J. Dommen, S. Henning, M. Ziese, A. Kiselev, F. Stratmann, I. George and U. Baltensperger, *Physical Chemistry Chemical Physics*, 2009, **11**, 8091-8097.
20. A. J. Prenni, M. D. Petters, S. M. Kreidenweis, P. J. DeMott and P. J. Ziemann, *Journal of Geophysical Research: Atmospheres*, 2007, **112**, D10223.
21. T. Tritscher, J. Dommen, P. F. DeCarlo, M. Gysel, P. B. Barmet, A. P. Praplan, E. Weingartner, A. S. H. Prévôt, I. Riipinen, N. M. Donahue and U. Baltensperger, *Atmos. Chem. Phys.*, 2011, **11**, 11477-11496.
22. J. Duplissy, P. F. DeCarlo, J. Dommen, M. R. Alfarra, A. Metzger, I. Barmpadimos, A. S. H. Prevot, E. Weingartner, T. Tritscher, M. Gysel, A. C. Aiken, J. L. Jimenez, M. R. Canagaratna, D. R. Worsnop, D. R. Collins, J. Tomlinson and U. Baltensperger, *Atmos. Chem. Phys.*, 2011, **11**, 1155-1165.
23. M. L. McGlashan, *Journal of Chemical Education*, 1963, **40**, 516.
24. C. B. Markwardt, Non-Linear Least Squares Fitting in IDL with MPFIT, 2008.
25. H. C. Price, B. J. Murray, J. Mattsson, D. O'Sullivan, T. W. Wilson, K. J. Baustian and L. G. Benning, *Atmos. Chem. Phys.*, 2014, **14**, 3817-3830.
26. B. Zobrist, V. Soonsin, B. P. Luo, U. K. Krieger, C. Marcolli, T. Peter and T. Koop, *Phys. Chem. Chem. Phys.*, 2011, **13**, 3514.
27. G. Hargreaves, N. O. A. Kwamena, Y. H. Zhang, J. R. Butler, S. Rushworth, S. L. Clegg and J. P. Reid, *The Journal of Physical Chemistry A*, 2010, **114**, 1806-1815.
28. K. L. Hanford, L. Mitchem, J. P. Reid, S. L. Clegg, D. O. Topping and G. B. McFiggans, *The Journal of Physical Chemistry A*, 2008, **112**, 9413-9422.
29. A. M. J. Rickards, R. E. H. Miles, J. F. Davies, F. H. Marshall and J. P. Reid, *The Journal of Physical Chemistry A*, 2013, **117**, 14120-14131.
30. J. F. Davies, A. E. Haddrell and J. P. Reid, *Aerosol Science and Technology*, 2011, **46**, 666-677.

31. B. J. Dennis-Smither, R. E. H. Miles and J. P. Reid, *Journal of Geophysical Research: Atmospheres*, 2012, **117**, D20204.
32. D. M. Murphy and T. Koop, *Quarterly Journal of the Royal Meteorological Society*, 2005, **131**, 1539-1565.
